# Immunotherapy of COVID-19 with poly (ADP-ribose) polymerase inhibitors: starting with nicotinamide

**DOI:** 10.1042/BSR20202856

**Published:** 2020-10-30

**Authors:** Abdulla A.-B. Badawy

**Affiliations:** Formerly School of Health Sciences, Cardiff Metropolitan University, Cardiff, Wales, U.K.

**Keywords:** Aryl hydrocarbon receptor, glucocorticoids, Indoleamine 2,3-dioxygenase, Kynurenine pathway, Nicotinamide, Poly (ADP-ribose) polymerase

## Abstract

COVID-19 induces a proinflammatory environment that is stronger in patients requiring intensive care. The cytokine components of this environment may determine efficacy or otherwise of glucocorticoid therapy. The immunity modulators, the aryl hydrocarbon receptor (AhR) and the nuclear NAD^+^-consuming enzyme poly (ADP-ribose) polymerase 1 (PARP 1) may play a critical role in COVID-19 pathophysiology. The AhR is overexpressed in coronaviruses, including COVID-19 and, as it regulates PARP gene expression, the latter is likely to be activated in COVID-19. PARP 1 activation leads to cell death mainly by depletion of NAD^+^ and adenosine triphosphate (ATP), especially when availability of these energy mediators is compromised. PARP expression is enhanced in other lung conditions: the pneumovirus respiratory syncytial virus (RSV) and chronic obstructive pulmonary disease (COPD). I propose that PARP 1 activation is the terminal point in a sequence of events culminating in patient mortality and should be the focus of COVID-19 immunotherapy. Potent PARP 1 inhibitors are undergoing trials in cancer, but a readily available inhibitor, nicotinamide (NAM), which possesses a highly desirable biochemical and activity profile, merits exploration. It conserves NAD^+^ and prevents ATP depletion by PARP 1 and Sirtuin 1 (silent mating type information regulation 2 homologue 1) inhibition, enhances NAD^+^ synthesis, and hence that of NADP^+^ which is a stronger PARP inhibitor, reverses lung injury caused by ischaemia/reperfusion, inhibits proinflammatory cytokines and is effective against HIV infection. These properties qualify NAM for therapeutic use initially in conjunction with standard clinical care or combined with other agents, and subsequently as an adjunct to stronger PARP 1 inhibitors or other drugs.

## Introduction

Understanding the immune effects of COVID-19 can pave the way for a rational choice of the appropriate immunotherapy. Huang et al. [1] reported that COVID-19 patients from Wuhan, China exhibit a proinflammatory environment characterised by elevated levels of plasma cytokines and chemokines, which is stronger in those requiring intensive care. A proinflammatory environment can lead to activation of the immune modulator the aryl hydrocarbon receptor (AhR) which in turn can activate the nuclear NAD^+^-consuming enzyme poly (ADP-ribose) polymerase 1 (PARP 1). PARP 1 activation leads to cell death by a mechanism involving in part depletion of NAD^+^ and adenosine triphosphate (ATP). Accordingly, targeting PARP 1 with inhibitors has been proposed [2–4] as a potential therapy of COVID-19. The latter three studies did not review evidence for modulation of the AhR by COVID-19, mainly because of the rapid advance in COVID-19 research currently taking place at an unprecedented pace. It should, however, be emphasised that the PARP1 reaction is important for DNA repair during an immune response and its activation is therefore not inherently pathophysiological, but can become so under certain conditions, e.g. when ATP and NAD^+^ are limiting.

In the present review, a detailed analysis of changes in cytokines and chemokines induced by COVID-19, other coronaviruses and the other conditions affecting lung function, the respiratory syncytial virus (RSV) and chronic obstructive pulmonary disease (COPD), will be made, evidence for up-regulation of the AhR in COVID-19 and other coronaviruses and that of PARP in RSV and COPD will be described, and a sequence of events culminating in cell death following COVID-19 infection will be proposed. Experimental and clinical studies addressing potential changes in the proposed sequence will be suggested. Finally, a review of some currently tested medications and of potential pharmacotherapies based on PARP 1 inhibition with particular emphasis on nicotinamide (NAM) will be made. This review is not intended to be exhaustive, but will be limited mainly to a discussion of issues related to the above aspects.

## Cytokine status of COVID-19 patients

Preliminary results [1] in 13 patients requiring and 28 not requiring intensive care and 4 healthy controls demonstrated a proinflammatory environment in COVID-19 patients. Whereas plasma levels of most cytokines and chemokines analysed were higher in patients than in controls (see Supplementary Figure S1 in [1]), a largely and stronger proinflammatory profile was observed in those requiring, compared with those not-requiring intensive care use (ICU) (Table 1 here). It would therefore be prudent not to aggravate this environment with use of immunosuppressants (see below). A second paper from China [5] examined cytokine and chemokine levels in a smaller number of COVID-19 patients: eight with severe and four with mild symptoms and eight control subjects. The authors confirmed the higher levels in severe- compared with mild-symptom patients of interleukin (IL)-2 (IL-2), IL-7, IL-12 and IL-17. Additionally, they reported higher levels of IL-1ra, IL-4, IL-10 and interferon γ (IFN-γ), whereas this group of cytokines were reported in the Wuhan study [1] to be comparable between ICU and non-ICU patients. Clearly, more detailed studies are required in larger patient and control samples. A more recent study from U.S.A. [6] examined cytokine profiles in a relatively larger sample of moderate (*n*=121) and severe (*n*=43) COVID-19 patients and healthy controls (*n*=43). The authors [6] identified a group of proinflammatory cytokines shared between mild and severe cases, namely IL-1α, IL-1β, IL-17A, IL-12 p70 and IFN-α and that, whereas levels of some of these declined with time in mild cases, raised levels of others, notably IFN-α, were maintained in severe cases, with IFN-α levels being the second largest death risk factor after IL-18. The greater elevation of IFN-α levels in severe cases was also reported in the second study from China [5] and the potential significance of this IFN-α elevation in relation to the efficacy of dexamethasone (DEX) in COVID-19 therapy will be discussed below.

**Table 1:** Pro- and anti-inflammatory cytokines in COVID-19 patients from Wuhan, China according to intensive care needs

ICU vs non-ICU-patients			
Higher proinflammatory	Similar proinflammatory	Higher anti-inflammatory	Similar anti-inflammatory
IL-2	IL-1β	IL-9	IL-1ra
IL-7	IL-6		IL-4
IL-8	IL-13		IL-5
IL-12	IFN-γ		IL-6
IL-17			IL-10
IP-10			
MCP-1			
MIP-1α			
MIP-1β			
TNF-α			
RANTES			

This table is derived from data in Supplementary Figure S1 of the article by Huang et al. [1] (Clinical features of patients infected with 2019 novel coronavirus in Wuhan, China. *Lancet* (2020); **395**, 497–506). Abbreviations: MCP, monocyte chemoattractant protein; MIP, macrophage inflammatory protein; TNF, tumour necrosis factor; RANTES, regulated on activation and normal T-cell expressed.

## Previous findings with coronaviruses and a pneumovirus

Plasma and tissue cytokine and chemokine concentrations were determined in patients with the coronaviruses severe acute respiratory syndrome (SARS) [7–9], Middle East respiratory syndrome (MERS) [10,11], the pneumovirus RSV [12] and in COPD patients [13]. The changes depicted in Table 2 show clearly that a proinflammatory environment also characterises these conditions. The increases in proinflammatory cytokines in plasma or serum of SARS and COPD patients occurred in subjects not requiring hospitalisation or intensive care, thus suggesting that, as with COVID-19, a proinflammatory environment exists in patients with these viral infections irrespective of severity. In one study in SARS patients [7], time-dependent differences in plasma cytokine changes following the onset of symptoms were observed, hence the need to perform time-course studies of plasma cytokine changes in COVID-19 patients as soon as possible after diagnosis. In the COPD study [13], the authors noted that the plasma changes in cytokines and chemokines are not seen in lung (broncho-alveolar lavage) material and suggested that the lung pathology originates elsewhere, or, alternatively, this material is not representative of the situation in lung tissue.

**Table 2:** Previous studies of cytokines and chemokines in SARS, MERS, RSV and COPD

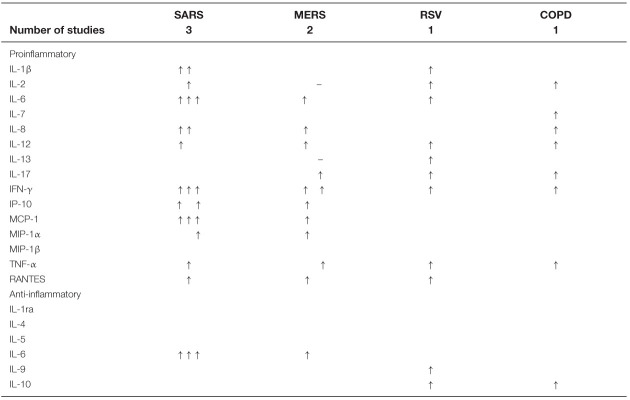

The symbols ↑ and – indicate respectively an increase above, or no change from, controls. Absence of a symbol indicates that a parameter has not been measured. The references to the studies quoted are as follows: SARS [7–9], MERS [10,11], RSV [12], COPD [13]. See the text for abbreviations and further comments.

Other aspects of immune function emerge from studies with a range of coronaviruses, the RSV pneumovirus and from those in COPD patients. Thus, activation of the AhR was demonstrated with SARS, MERS, COVID-19 [14], with the mouse hepatitis virus (MHV) model [15] and the pneumovirus RSV [16]. MHV activates the AhR independently of the extrahepatic tryptophan (Trp)-degrading enzyme indoleamine 2,3-dioxygenase (IDO) [15], causing cytokine modulation and inducing expression of PARP, and RSV-infected mesenchymal stem cells regulate immunity via IFN-β and IDO [16]. PARP 1 is activated in COPD patients [17]. These findings, some of which are recent, coupled with the important observation that the AhR regulates the PARP 1 gene [18] provide a justification for targeting PARP 1 (and possibly also the AhR) for COVID-19 therapy.

## The AhR

The ligand-activated transcription factor AhR can elicit both protective and destructive influences on immune responses. In general, endogenous ligands activate the AhR in a manner that prevents excessive inflammation and autoimmunity, whereas exogenous ligands enhance inflammatory responses to infection, resulting in a state of pathological immunosuppression [19,20]. 2,3,7,8-Tetrachlorodibenzo-*p*-dioxin (TCDD), a high-affinity exogenous ligand, induces a heightened inflammatory response upon binding to the AhR that can elicit pathological changes in lung, gut and skin [20]. Other exogenous AhR ligands include other halogenated aromatic hydrocarbons, such as dibenzofurans and biphenyls, polycyclic aromatic hydrocarbons, such as 3-methylcholanthrene, benzo-[*a*]-pyrene, benzanthracenes and benzoflavones, indirubin and 6-formylindolo(3,2-*b*) carbazole. Some of these are short-acting, because of their rapid metabolic clearance, unlike TCDD, whose effects last for days, rather than only a few hours, because of its poor metabolism by virtue of its four Cl atoms impeding access by metabolising enzymes [19].

**Figure 1:**
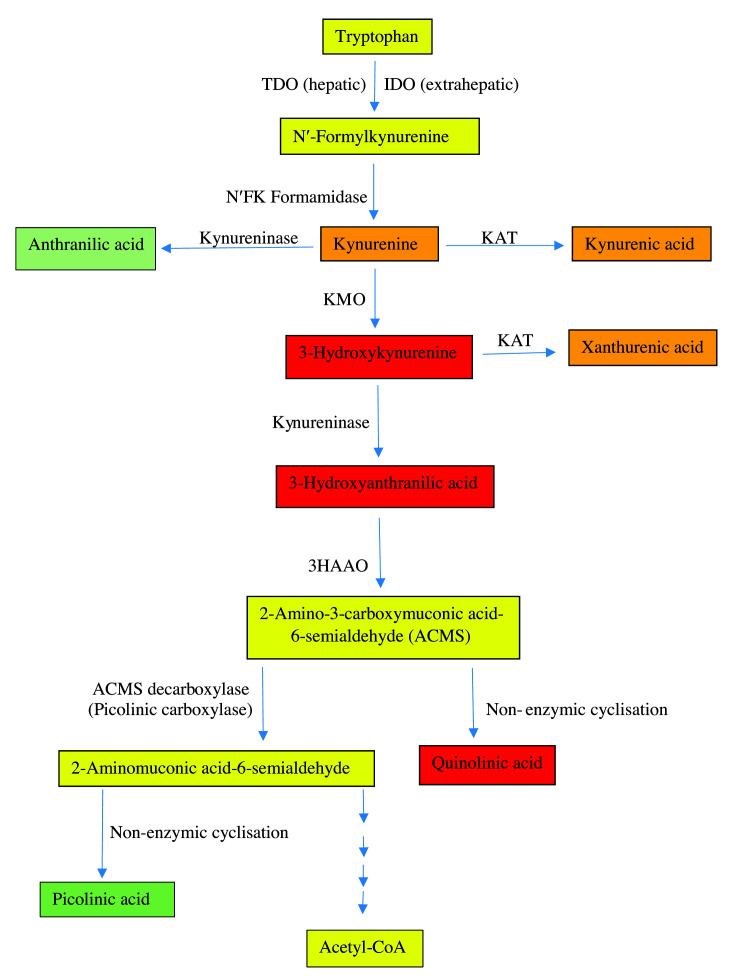
The Kyn pathway of tryptophan degradation Adapted from Figure 1 in [26] [Badawy, A.A.-B. (2017). Kynurenine pathway of tryptophan metabolism: regulatory and functional aspects. Int. J. Tryptophan Res. 10, 1-20, doi: 10.1177/1178646917691938]. Colours indicate a proinflammatory (red), anti-inflammatory (green), a mixed or partially proinflammatory (amber) or a normal metabolite (yellow). Abbreviations: 3-HAAO, 3-hydroxyanthranilic acid 3,4-dioxygenase; KAT, Kyn aminotransferase; KMO, Kyn monooxygenase; N′FK, N′-formylkynurenine; TDO, tryptophan 2,3-dioxygenase.

Endogenous AhR ligands include Trp metabolites formed in the kynurenine (Kyn) pathway (KP) (Figure 1). Since demonstration of Kyn as an endogenous Trp metabolite AhR ligand [21], it has generally been assumed that Kyn is the sole ligand. However, studies with other Kyn metabolites established that kynurenic acid (KA) has the greatest affinity for the AhR, followed by xanthurenic acid (XA), with Kyn itself being a much weaker ligand [22]. These latter authors suggested that KA can activate the AhR at physiologically relevant concentrations. They showed that KA enhances the expression of CYP1A1 mRNA in HEP-G cells significantly at a 100 nM concentration that is approximately three-fold higher than the normal plasma level of 31 nM quoted. A 100 nM concentration (representing the KA IC_25_ or the concentration that can achieve 25% of AhR activation by 10 nM TCDD [22]) can be reached easily if [Kyn] is increased moderately. For example, a ∼50% increase in human plasma [Kyn] following a small dose oral Trp load (1.15 g to a ∼70 kg adult) results in a three-fold increase in [KA] [23].

AhR activation in viral infection must involve participation of an endogenous ligand(s). As stated above, MHV activates the AhR by an IDO-independent mechanism [15]. As KA appears to be the major endogenous AhR ligand among Kyn metabolites, it is possible that this mechanism can still be mediated by KA produced from Kyn by other mechanisms, such as up-regulation of liver Trp 2,3-dioxygenase (TDO) and/or increased flux of plasma free Trp down the KP (see below). As will be stated below, another source of KA is the Trp transamination product indol-3-ylpyruvic acid (IPA). The possibility of increased flux of plasma free Trp in viral infections will be considered below.

A KA-mediated activation of the AhR is not a simple process. While details of the complex interactions underlying this process are outside the scope of this article, it is important to emphasise here that IDO expression is controlled by the AhR through an autocrine loop involving AhR-IL-6-STAT3 signaling [24,25] and that, while KA can induce IL-6 production, IL-6 generated by inflammation can induce IDO to produce sufficient amounts of KA to activate the AhR [22].

## The Kyn pathway of tryptophan metabolism

**Figure 2:**
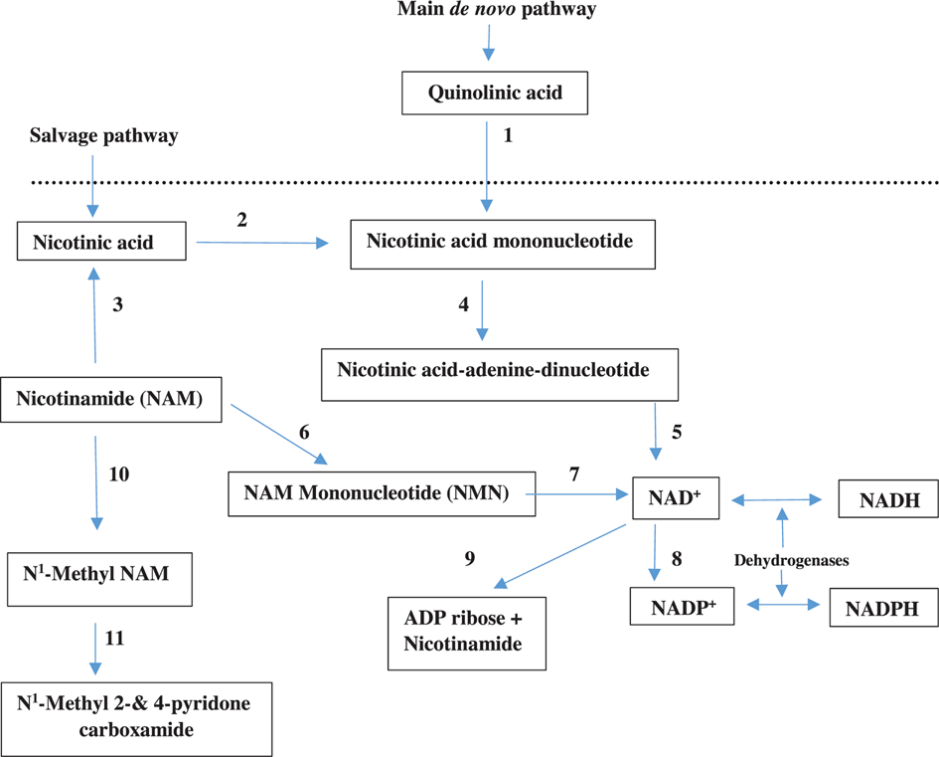
NAD^+^ synthesis from quinolinic acid in the *de novo* pathway and from nicotinic acid and NAM in the salvage pathway Adapted from Figure 2 in [26] [Badawy, A.A.-B. (2017). Kynurenine pathway of tryptophan metabolism: regulatory and functional aspects. Int. J. Tryptophan Res. 10, 1-20, doi: 10.1177/1178646917691938]. Enzymes are numbered as follows: **1**, quinolinic acid phosphoribosyltransferase; **2**, nicotinic acid phosphoribosyltransferase; **3**, nicotinamide deaminase; **4**, nicotinic acid mononucleotide adenylyltransferase; **5**, NAD synthetase; **6**, nicotinamide phosphoribosyltransferase; **7**, nicotinamide mononucleotide adenylyltransferase; **8**, NAD kinase; **9**, NADase; **10**, nicotinamide N-methyltransferase; **11**, aldehyde oxidase. Abbreviations: ADP, adenosine diphosphate; NAD^+^(P^+^)H, oxidised and reduced nicotinamide-adenine-dinucleotide (phosphate).

The KP is the major Trp-degradative pathway, accounting for ∼95% of dietary Trp metabolism [26]. Under normal physiological conditions, the liver is the major site of the KP, being responsible for ∼90% of Trp degradation, with the remaining 5% occurring elsewhere, including immune cells. However, during immune activation, the extrahepatic KP assumes a greater quantitative significance in Trp degradation. The KP is controlled mainly by TDO in liver and IDO extrahepatically. As shown in Figure 1, Kyn undergoes mainly oxidative metabolism, to 3-hydroxykynurenine (3-HK) by Kyn monooxygenase and then to 3-hydroxyanthranilic acid (3-HAA) by kynureninase. This latter enzyme can also hydrolyse Kyn to anthranilic acid (AA). 3-HAA is further oxidised by its dioxygenase (3-HAAO) to an unstable intermediate: 2-amino-3-carboxymuconic acid-6-semialdehyde (ACMS), which occupies a central position at two junctions of the pathway. The pathway favours the non-enzymic cyclisation of ACMS to quinolinic acid (QA) and subsequent synthesis of NAD^+^. The minor junction involves decarboxylation of ACMS by ACMS decarboxylase (picolinate carboxylase) to form 2-amino-3-muconic acid-6-semialdehyde (AMS), which can undergo either non-enzymic cyclisation to picolinic acid (PA) or further metabolism to acetyl-CoA. Kyn and 3-HK can also be transaminated to KA and XA respectively in a minor reaction catalysed by Kyn aminotransferase (KAT). KA and XA formation, however, is limited by availability of the respective substrates in view of the high *K*_m_ of the enzyme [26]. Thus, formation of the AhR ligands KA and XA is dependent on [Kyn]. Details of NAD^+^ synthesis from QA in the main (*de novo*) pathway and the ‘salvage’ pathway from nicotinic acid and NAM are illustrated in Figure 2. Trp is a more effective source of NAD^+^ synthesis via QA in the *de novo* pathway than NAM or nicotinic acid in the salvage pathway (see [26] for review and references).

The KP produces a range of metabolites that are biologically active at various physiological levels and body systems [26–28]. Of particular relevance to COVID-19 are the immunomodulatory properties of some KP metabolites, namely the proinflammatory 3-HK, 3-HAA and QA, the anti-inflammatory PA and the dually acting KA. Increased production of these metabolites can be achieved by a number of mechanisms: TDO induction by glucocorticoids, IDO induction by IFN-γ, flux of plasma free (non-albumin-bound) Trp down the pathway and up-regulation of KMO and subsequent enzymes. Raising levels of proinflammatory Kyn metabolites by whatever intervention or mechanism may aggravate the immunosuppressive environment of COVID-19 to a greater pathological level. IFN-γ, however, has been shown [29] to be required for viral clearance in a mouse model of coronavirus retinopathy, the JMH strain of the MHV. The authors, however, suggested that IFN-γ acts by a variety of mechanisms and plays an important role in ocular inflammatory disease irrespective of the presence or absence of viral infection. As will be discussed below, the MHV model appears to exhibit a major increase in the flux of plasma free Trp down the KP, such that hepatic [Trp] and [Kyn] are strongly elevated. Under these conditions, the presence of high levels of Trp (>10 µM) can help maintain T-cell proliferation and prevent immune suppression by IFN-γ-stimulated IDO induction of Kyn metabolites [30,31], possibly allowing IFN-γ to act by the other mechanisms suggested for this particular experimental retinopathy model.

## The PARPs

The PARPs family of enzymes catalyse the transfer of adenosine diphosphate (ADP)-ribose to target proteins, thereby influencing many important processes, including DNA repair. PARP 1 activation can result in a multifaceted programmed cell death pathway involving in part depletion of NAD^+^ and ATP [32,33]. Thus, PARP 1 overactivation occurs in a number of cancers and can be induced by irradiation and in fibroblasts by DNA alkylating agents or by H_2_O_2_ leading to necrotic cell death [32,33]. As will be discussed below, PARP 1 activation can also produce an anti-inflammatory response. Thus, while PARP 1 activation may generally be protective, when the enzyme is overexpressed, its inhibition is metabolically favourable [34]. Preclinical data suggest that PARP inhibitors may be effective therapies for inflammatory, metabolic and neurological disorders [35]. The deleterious effects of PARP activation are further emphasised by observations in patients with lung diseases. Thus, COPD is associated with activation of systemic PARP 1 in peripheral blood lymphocytes [17] and the Ala allele polymorphism in the *PARP1* gene is associated with a decreased risk of asthma [36]. Also, the PARP 1 inhibitor olaparib protects against various aspects of lung dysfunction in the elastase-induced mouse model of COPD [37].

## Additional findings of relevance to the immune effects and therapy of COVID-19

### AhR activation and enhanced PARP1 expression by MHV

In the study with the coronavirus MHV mouse model [15], the AhR is activated by a mechanism(s) independent of IDO1 induction. The question arises if IDO induction by administration of inducers such as IFN-β, which is being trialled in COVID-19 patients, will additionally activate the AhR, thus further enhancing PARP 1 expression (see below). A potential AhR activation by IFN-β is likely to be additive to that by COVID-19, if the two act by different mechanisms. Embarking on IFN-β therapy should therefore be approached with caution. If AhR activation by COVID-19 is also IDO-independent, it is possible that it could still involve production of Kyn metabolites by other mechanisms, such as glucocorticoid induction of liver TDO or increased flux of plasma free Trp through TDO and down the KP (see below).

### Expressions of IFN-β and IDO in RSV infection

RSV infected mesenchymal stem cells exhibit a 100-fold increase in IFN-β expression and a 70-fold increase in IDO, and neutralising IFN-β reverses the increased IDO expression and activity [16]. Up-regulation of these two expressions was suggested by these latter authors to modulate the immune regulatory function, causing impairment of immune cell proliferation, which may account for the lack of protective RSV immunity and for chronicity of RSV-associated lung diseases such as asthma and COPD. The authors, however, did not observe a difference in viral load or increased IFN-β expression between wildtype and IDO KO mice and suggested that this mouse model does not reflect the human disease. Other studies with RSV patients did not report on IFN-β (which may not have been overexpressed), but observed enhanced expression of a wide array of cytokines and chemokines in the lower and upper respiratory tracts (see [38] for a review). Levels of IFN-β and IDO expression therefore need to be measured in COVID-19 patients. A significant increase in IFN-β in COVID-19 would not justify the use of this cytokine as a therapy, unless when targeted directly to the lungs by inhalation, though this will not prevent its rapid entry into the bloodstream. A preliminary report by the Company Synairgen given to the popular press suggests that inhalation of its IFN-β product reduces the risk of severe illness, the need for O_2_ and the time to discharge from hospital. The study population is presumably that of patients mainly with mild symptoms.

## Hypothesis: Sequence of events with COVID-19

**Figure 3:**
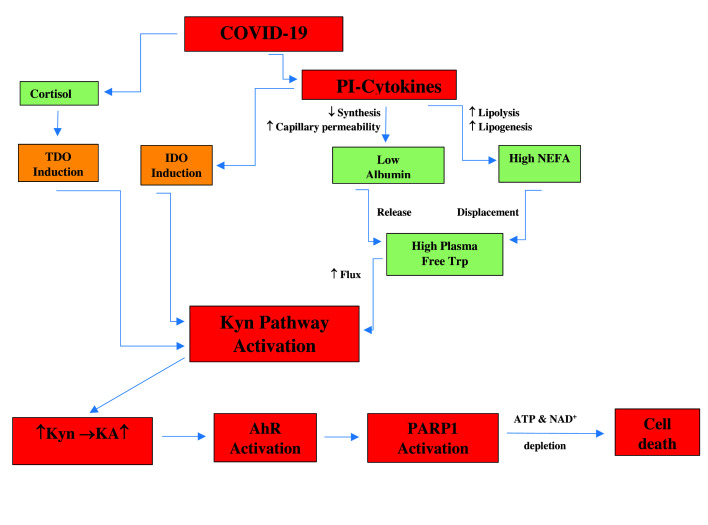
Diagrammatic representation of known and likely changes in immune function and tryptophan-metabolic parameters in COVID-19 For details and comments, see the text. Colours indicate a serious change (red), an intermediate change (amber) and a primarily normal physiological change (green). Abbreviations: NEFA, non-esterified fatty acids PARP, poly (adenosine-diphosphate-ribose) polymerase; PI, proinflammatory; TDO, tryptophan 2,3-dioxygenase; Trp, tryptophan.

A hypothesis is graphically illustrated in Figure 3. A sequence of events is proposed of the likely effects of COVID-19 culminating in cell death. Activation of PARP 1 is the final step preceding cell death as a result of depletion of both NAD^+^ and ATP. Lung function is especially vulnerable to impaired mitochondrial oxidative energy metabolism and it is therefore very likely that PARP 1 may play a central role in COVID-19-induced mortality. So far, the increase in plasma proinflammatory cytokines, the overexpression of the AhR and the decrease in albumin (see below) are the only known changes induced by this coronavirus. All other changes listed in Figure 3 are therefore hypothesis-driven, but based on observations in other viral infections, and can be assessed experimentally. The present hypothesis places Trp metabolism along the KP at the heart of COVID-19 pathophysiology.

## What needs to be measured in COVID-19 patients?

### Immune modulators and the tryptophan-metabolic status

In addition to assessing the expressions of the AhR, PARP1, IFN-β and IDO in suitable peripheral blood mononuclear cells and of plasma pro-and anti-inflammatory cytokines in larger patient and control samples, the Trp-metabolic status of COVID-19 patients should be established through measurements of fasting plasma Trp, Kyn and the Kyn metabolites 3-HK, 3-HAA, KA and QA. Levels of most of the above are likely to be elevated in patients, especially in severe cases. Only plasma total [Trp] may be decreased with IDO/TDO induction or changes in albumin and non-esterified fatty acids (NEFA) inducing Trp displacement from albumin-binding sites and increasing its flux down the KP (see below). Changes in IFN-β levels cannot be predicted from current information.

### Cortisol

The other Trp-degrading enzyme, liver TDO, may also be involved through glucocorticoid induction if cortisol levels are elevated in COVID-19. Activation of the hypothalamic-pituitary-adrenal (HPA) axis resulting in increased release of cortisol is a feature of the stress response to viral (and bacterial) infections [39]. The cortisol status in COVID-19 infection is unclear and a prospective study is currently underway [40]. Raised cortisol levels are observed in RSV and Ebola patients and in SARS patients with lymphopaenia [41]. In the Wuhan study [1], lymphopaenia was significantly more predominant (85%) in patients requiring intensive care, compared with those not requiring it (54%), though numbers were small. It is therefore possible that cortisol will be raised in a proportion of COVID-19 patients irrespective of severity. In such cases, TDO induction by cortisol is likely to elevate the availability of Kyn to subsequent enzymes of the KP and thus promote the formation of proinflammatory Trp metabolites [26]. Whereas TDO expression requires access to hepatic tissue, enhanced TDO activity results in reciprocal changes in plasma [Trp] and [Kyn], though this can also reflect increased flux of plasma free Trp through TDO. A decrease in plasma free and total [Trp] characterises TDO induction, whereas an increase in free and a decrease in total [Trp] reflect increased Trp availability secondarily to displacement from albumin-binding sites [28] (see below).

### Plasma-free Trp and determinants of its flux

Another determinant of Kyn formation is the flux of plasma-free (non-albumin-bound) Trp through TDO and down the pathway, without the need for TDO/IDO induction [26]. The bulk of plasma [Trp] is albumin-bound, with only 5–10% freely circulating. Free [Trp] is therefore controlled by concentrations of the binder albumin and the physiological displacers NEFAs. In the Wuhan study [1], [albumin] (median and range in g/l) was 27.9 (26.3–30.9) in intensive care patients and 34.7 (30.2–36.5) in non-intensive care patients. No control values were given. However, as the normal range is 35–50 g/l, the value for non-intensive care patients approaches the normal range. Consequently, intensive care patients can be assumed to have at least 20% less albumin. A decrease in [albumin] of ≥19% can significantly cause the release of bound Trp [28]. Serum [NEFA] has not been measured in COVID-19 patients, but is known to be elevated in infections with other viral classes, e.g., the flavivirus Dengue [42], the lentivirus HIV [43] and the asfivirus, the swine (fever) influenza [44]. Albumin depletion coupled with NEFA elevation provide the maximum effect on Trp binding and the resultant increase in free Trp availability can enhance the production of Kyn through IDO/TDO and thus provide the substrate for proinflammatory KP metabolite formation [26]. In situations involving strong and sustained displacement of albumin-bound Trp, the associated increase in tissue uptake of the free amino acid coupled with the rapid equilibration between the free and bound fractions results in a decrease in plasma total [Trp] [27], which should not be interpreted to indicate Trp depletion, but, rather, increased utilisation. Measurements of plasma-free [Trp] along with albumin and NEFA in COVID-19 patients can be informative. The mouse MHV model is the first coronavirus to be shown to induce simultaneous increases in liver [Kyn] and [Trp] [45] that are consistent with increased flux of circulating free Trp through liver TDO and down the KP [46].

## Potential therapies of COVID-19

### Existing drugs in current trials

Until vaccine therapy becomes available, clinicians are currently trialling various therapies previously used in viral and other diseases. These include antiviral, immunomodulatory, anti-inflammatory and miscellaneous drugs. A comprehensive review of the current status of these trials has been published [47] and examples of these therapies will be discussed briefly here, in particular, Remdesivir, the antimalarial drugs chloroquine and hydroxychloroquine, glucocorticoids, IFN-β, IL-6 antagonists and angiotensin-converting enzyme 2 (ACE 2) inhibitors.

#### Remdesivir and antimalarials

With Remdesivir, efficacy is partial, though valuable and much needed: it does not lower death rates, but reduces the time to recovery by ∼27% [48,49]. With the antimalarials chloroquine and hydroxychloroquine, efficacy is unproven, results of clinical trials are equivocal and the drugs possess serious potential side effects, especially in severely ill patients [50].

#### Glucocorticoids

With corticosteroids (glucocorticoids), current evidence does not justify their use and the WHO does not recommend them for COVID-19 therapy [51]. Glucocorticoids can compromise antiviral defences and enhance respiratory viral and other infections, especially when given in high doses and/or for long durations [52–54]. Additionally, glucocorticoids could increase production of proinflammatory Kyn metabolites by TDO induction and thus aggravate the proinflammatory environment of COVID-19 patients. One glucocorticoid, dexamethasone (DEX), however, has been shown to decrease the death rate of COVID-19 patients in a recent trial [55]. The rate reduction in ventilated patients was ∼30%, whereas that in patients on O_2_ only was ∼20%. No benefit was accrued by patients not requiring respiratory support. Thus, DEX appears to be effective only in COVID-19 patients with severe symptoms.

How does DEX differ from other glucocorticoids, apart from being the strongest anti-inflammatory [56] and a much stronger TDO inducer than cortisol [57,58]? Whereas these differences may not explain the efficacy of DEX in COVID-19, other differences provide potential explanations, in particular, effects on IDO induction by IFNs and inhibition of prostaglandin biosynthesis. Thus, IFN induction of IDO is inhibited much more strongly by DEX than by cortisone (IC_50_ = 1 nM for DEX and 20 nM for cortisone) [59]. This strongly suggests that IDO induction may play an important role in COVID-19 patients, as suggested in Figure 3 here, especially in those with severe symptoms requiring intensive care, and, by inference, IDO-dependent production of proinflammatory Kyn metabolites may be a vital component of the pathophysiology of this viral infection. In the above study [59], the type of IFN was not defined. DEX at 10 nM inhibits IDO induction by IFN-α by 71%, but potentiates that by IFN-γ by two-fold in human peripheral blood monocytes [60]. IFN-γ-induced IDO activity potentiated by DEX is 28-fold greater than the IFN-α-induced IDO activity inhibited by DEX. Does this suggest that DEX could be effective in COVID-19 patients with raised IFN-α, but without benefit or even harmful in those with raised IFN-γ? Evidence in support of this possibility is provided by the finding in the larger COVID-19 study mentioned above [6] that patients with severe symptoms exhibit a sustained elevation of plasma IFN-α, whereas those with mild symptoms show a moderate decline. The importance of IFN-α in COVID-19 pathology is further emphasised by the observation [6] that this cytokine is the second largest death risk factor after IL-18, a cytokine referred to as the ‘IFN-γ-inducing factor’. By contrast, IFN-γ remains unaltered in severe cases, but declines sharply in moderate ones [6]. Perhaps the balance between the α- and γ-IFNs determines DEX efficacy and could be assessed as a possible predictor of response to therapy with DEX. IFN-γ is the strongest IDO inducer among IFNs, followed by IFN-β, with IFN-α being the weakest inducer [61]. Though the weakest, IFN-α is strong enough to induce Trp degradation along the KP [62,63].

**Figure 4:**
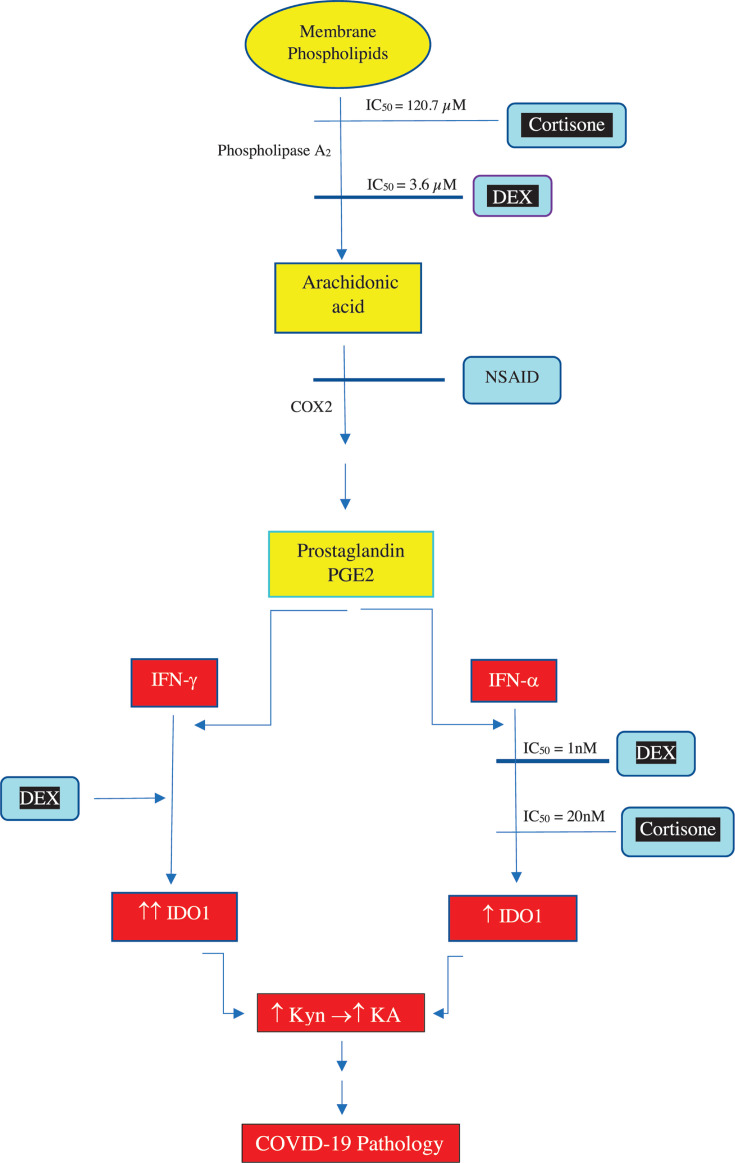
Differential effects of DEX and cortisone on inflammatory mediators explaining their likely pharmacotherapeutic differences in COVID-19 Colours indicate a normal metabolic pathway (yellow), glucocorticoids and NSAID (blue) and agents causing harm (red).

The efficacy of DEX in COVID-19 therapy may be related to prostaglandin metabolism and actions. Of the complex interrelationships between prostaglandins (PGs) and inflammation [64], little attention has been paid to the specific interaction of these immune modulators with Trp metabolism. Thus, IDO induction by IFNs involves participation of PG biosynthesis and one enzyme involved in this biosynthesis, phospholipase A_2_, exhibits different sensitivities to inhibition by glucocorticoids, with DEX being a much stronger inhibitor than cortisone (IC_50_ = 3.6 µM for DEX and 120.7 µM for cortisone) [59]. A detailed discussion of the relationships between and interactions of cytokines and prostaglandins is outside the scope of this article, but a few relevant features are noteworthy. Thus: (1) IFNs enhance the synthesis and activation of phospholipase A_2_ [65,66]. (2) As well as by non-steroidal anti-inflammatory drugs (NSAID), another enzyme of PG biosynthesis, cyclooxygenase isoform 2 (COX2), is also inhibited by adrenal glucocorticoids [67,68] and by DEX [69]. A simple diagram summarising differences between DEX and cortisone that may explain the efficacy of DEX in COVID-19 patients is presented in Figure 4. As the diagram suggests, IDO1 and hence KP activity are likely to feature prominently in COVID-19 pathophysiology.

Two case studies reported favourable responses of COVID-19 patients to the other powerful synthetic glucocorticoid methylprednisolone: a case of a renal transplant recipient [70] and three severe cases who did not respond to therapy with the IL-6 inhibitor tocilizumab [71]. The latter three cases showed a rapid improvement with pulse administration of a high daily intravenous dose of methylprednisolone of 1 g for 3 days and intravenous immunoglobulin. However, a recent placebo-controlled randomised clinical trial of short-term (7-day) adjunctive methylprednisolone therapy failed to demonstrate a robust efficacy [72]. Thus, whereas the overall death rate was not altered, patients aged > 60 years had a lower death rate by day 28. A current trial reported in the popular press [73] demonstrated efficacy of cortisol in severe cases with COVID-19 infection. These studies underscore the need to perform detailed investigations into similarities and differences within and between synthetic and natural glucocorticoids on aspects of tryptophan and prostaglandin metabolism that impact immune function as illustrated by the above findings.

#### IFN-β, IL-6 antagonists and ACE 2 antibodies

IFN-β is currently being trialled and unpublished results announced in news media suggest that it exerts the beneficial effects described above. Little is known about the effects of this IFN on prostaglandin biosynthesis or any likely modulation thereof by glucocorticoids. As far as I could ascertain, the status of IFN-β in COVID-19 is unknown. A number of IL-6 antagonists are also being trialled, including tocilizumab [71,74,75]. Small numbers of patients were examined to draw firm conclusions [71,74] and, whereas it is effective in some patients and can reduce the risk of mechanical ventilation and death, this drug can also induce new infections [75]. Inhibition of angiotensin-converting enzyme, ACE 2, has also been suggested. Whereas recent discussions have centred on potential sensitivity of ACE 2 inhibitor-treated patients to COVID-19 [76], clinical trials of ACE 2 antibodies have been suggested [77].

### Therapies countering proposed effects

Effort can be devoted to assessing the changes proposed in Figure 3 and, if demonstrated, counter measures could be suggested as follows: TDO and IDO induction can be blocked with glucocorticoid antagonists and IDO inhibitors respectively. Increased flux of plasma-free Trp down the KP can be minimised by albumin infusions, antilipolytic agents or both. These measures can minimise excessive production of proinflammatory Kyn metabolites and potentially also activation of the AhR and consequently that of PARP1.

### PARP 1 inhibitors

In the short term, efforts should focus on the distal changes in the proposed sequence of events in Figure 3, in particular PARP 1. PARP 1 inhibition will most likely prevent cell death due to NAD^+^ and ATP depletion. PARP 1 inhibition has been suggested using NAM [4] or CVL218 (Mefuparib) [3]. This latter compound inhibits SARS viral replication with an IC_50_ of 5.12 µM, and IL-6 production by activated peripheral blood mononuclear cells [3]. As stated earlier, another PARP 1 inhibitor, olaparib, protects against lung dysfunction in a mouse model of COPD [37]. As will be described below, PARP 1 inhibition by Mefuparib is much stronger than that by NAM.

#### NAM: an ideal profile for PARP 1 inhibition

While various PARP inhibitors are currently being trialled in other conditions [35], a more readily available inhibitor is the NAD^+^ precursor NAM [78,79] (and not nicotinic acid). By inhibiting PARP 1 and restoring ATP levels as well as acting as NAD^+^ precursor, NAM possesses the desired profile for a potential effective therapy of COVID-19. Examples of its favourable profile include abrogation of acute lung injury caused by ischaemia/reperfusion [80], inhibition of proinflammatory cytokines [78] and efficacy against HIV infection (see [81] for a review).

NAM possesses the following unique advantages regarding ATP, NAD^+^ and PARP. (*i*) It inhibits PARP 1 activity by competing with NAD^+^ for the enzyme active site. (*ii*) As a result, it conserves NAD^+^ and (*iii*) restores ATP levels. (*iv*) As the NAD^+^ precursor, it further increases its levels. (*v*) The increased concentration of NAD^+^ provides the substrate for NAD^+^ kinase, leading to production of NADP^+^, which is a stronger PARP inhibitor [82], thereby strengthening the PARP 1 inhibitory capacity. (*vi*) NADP^+^ which acts by competing with NAD^+^ thus conserving NAD^+^ levels, is able to inhibit various classes of PARP other than PARP1 [82]. (*vii*) NAM is also able to inhibit non-competitively another NAD^+^-consuming enzyme, Sirtuin 1 (SIRT 1: silent mating type information regulation 2 homologue 1) at physiological concentrations (IC_50_ < 50 µM) [83], thereby further conserving NAD^+^ levels. Other relevant and important effects of NAM further supporting its use in COVID-19 therapy will be described below.

PARP 1 inhibition by NAM, however, is a weak one: a 50% inhibition at 0.5 mM [79], in contrast with the strong one by CVL218 (IC_50_ = 3.2 nM) [84]. However, NADP^+^, which has been proposed to be the endogenous PARP 1 inhibitor, is a stronger inhibitor than NAM, demonstrating inhibition at 10 µM [82]. Perhaps the weak inhibition by NAM in cell systems *in vitro* may be due to its slow conversion into NADP^+^. The study by Bian et al. [82] showed that: (1) PARP 1 recognises NADP^+^, but does not use it as a substrate; (2) the ratio of [NADP^+^]/[NAD^+^] is an important determinant of PARP 1 inhibition, with a modest increase producing a significant inhibition; (3) NAD(P)H does not inhibit PARP activity. That NAM administration can increase [NADP^+^] has been demonstrated in rat liver [85]. When given in drinking water at a concentration of 100 mg/l, resulting in a daily dose of 10–15 mg/kg body weight, NAM increases hepatic [NADP^+^] by 42–60% and [NADPH] by 26–30% respectively. With a normal rat liver [NADP^+^] of ∼54 µM [86], the above increase by NAM raises it to ∼80 µM. As NADP^+^ is formed from NAD^+^, it is almost certain that the [NADP^+^]/[NAD^+^] ratio will have been increased by NAM. Given that, compared with humans, rodents generally require doses of chemicals a few orders of magnitude higher, the NAM dosage needed to inhibit PARP 1 in COVID-19 patients is unlikely to be excessive. In humans, doses of NAM of up to ∼3 g daily (equivalent to ∼40 mg/kg for a 70 kg adult) are generally acceptable, with higher doses requiring supervision [87]. As a nutritional ‘vitamin-like’ substance, NAM should, if required, receive rapid approval from Ethics Committees and regulatory authorities.

A comparison of the effects of NAM and Mefuparib may be useful. The only clear advantage of Mefuparib over NAM is its strong PARP 1 inhibitory potency. Both decrease IL-6 production and conserve NAD^+^ and ATP. However, only NAM will cause an additional increase in NAD^+^ and increased synthesis of NADP^+^. Finally, whereas NAM has been shown to possess a favourable profile in a number of inflammatory states, Mefuparib has not as yet undergone corresponding studies other than inhibition of viral replication [3]. Thus, given the urgent need to address the COVID-19 pandemic, the use of NAM can be initiated before current and future clinical trials and experimental studies with the stronger PARP 1 inhibitor Mefuparib and other strong inhibitors establish their safety and other requirements and efficacy in COVID-19 patients. Even if stronger PARP 1 inhibitors are proven to be effective in COVID-19 patients, NAM, by virtue of its additional effects, especially those of enhanced NAD(P)^+^ synthesis, can be used as an adjunctive therapy.

A note of caution is appropriate here. In a mouse model of acute lung injury induced by mechanical ventilation, NAM was reported [88] to cause hypoxemia, yet no such effect of NAM was observed in other studies referenced by these authors [88] with other models of inflammatory diseases, including acute lung injury, in mice, rats and hamsters. As stated above, NAM abrogates lung injury caused by ischaemia/reperfusion [80]. Jones et al. [88] did not specify the mouse strain(s), which included the C57, used in their various experiments. Mouse strains are known to differ in their response to immune insults, with the C57BL being the most vulnerable [89]. However, clinicians should nevertheless consider this possibility by monitoring blood O_2_ levels during clinical management when using NAM.

It is also important to emphasise other aspects of NAM actions that are relevant to the proposed PARP 1 inhibition therapy of COVID-19. NAM, as ‘vitamin’ B_3_, has been suggested [90] to help maintain homoeostasis partly through the involvement of gut microbiota and that the proinflammatory environment of COVID-19 along with oxidative stress can undermine this process. The complex relationships of, and interactions between, NAM, NAD^+^, PARP 1 and SIRT 1 are fascinating and worthy of discussion (for references, see [91]). Thus, PARP 1 and SIRT 1 antagonistically regulate one another by virtue of competition for their common co-substrate NAD^+^. Accordingly PARP 1 activation inhibits SIRT 1 through generation of NAM, which is a potent SIRT 1 inhibitor [83,92], whereas PARP 1 is inhibited when SIRT 1 is activated by the NAM metabolite 1-methylnicotinamide or after treatment with NAM riboside (NAMR). NAM therefore can activate PARP 1 and potentiate that by resveratrol, though in several respects, it alone exhibits a similar or greater ability to undermine proinflammatory cytokine gene expression and secretion by macrophages than resveratrol [91]. Thus, the antiinflammatory activity of NAM does not involve SIRT 1, but could be mediated by PARP 1 induction of BCL6 (B Cell lymphoma 6 protein) and concomitant inhibition of COX2 [91,93,94].

## General discussion and conclusions

The present discussion also raises important points regarding the role of NAD^+^ and its precursors in maintenance of immune function and their ability to combat COVID-19 infection, the possible determination of glucocorticoid efficacy or otherwise by the proinflammatory profile induced by COVID-19 and the mechanisms of actions of prostaglandins.

The role of NAD^+^ in the maintenance of homoeostasis and integrity of the immune system is an undisputed fact [95]. In infection, the increased demand for NAD^+^ is necessitated by a variety of changes, including enhanced activities of PARP 1, SIRT 1, NAD^+^ hydrolase and cell surface ADP ribosyl transferases [95,96]. In COVID-19 infection, it is more likely that efforts at replenishing NAD^+^ levels will be more important than PARP 1 inhibition *per se*, at least under conditions involving moderate activation of PARP 1. PARP 1 inhibition will, however, be an important requirement if the enzyme is overactivated, particularly in severe cases, but this remains to be assessed in future studies.

NAD^+^ synthesis is achieved mainly from dietary Trp in the *de novo* biosynthetic pathway, rather than via the Preiss-Handler ‘salvage’ pathway from NAM or nicotinic acid, with quinolinic acid playing an important role [26,95,96]. A comparison of the relative contributions of these three NAD^+^ precursors and also the NAM derivatives NAM riboside (NAMR) and reduced NAMR (NAMRH) to increased NAD^+^ availability may help refine the choice of the most appropriate precursor for COVID-19 therapy. Although Trp is a more efficient NAD^+^ precursor than nicotinic acid or NAM [97–99], its use cannot be recommended in COVID-19, as it is likely to further increase the production of proinflammatory Kyn metabolites over and above that induced by cytokine induction of IDO1 activity. Even if exogenous Trp inhibits IDO1 activity if levels above 100 µM are achieved, which is the case after loading with a small 1.15 g Trp dose in humans [23], the increased flux of plasma-free Trp through the KP can override the IDO inhibition. Furthermore, exogenous Trp can undergo greater transamination to indol-3-ylpyruvic acid (IPA), which, as will be described below, is a strong PARP activator. NAM administration to mice with focal ischaemia reverses the decrease in [NAD^+^] in cerebral cortical structures and increases it by up to ∼2.4-fold [92]. As stated above, NAM inhibits PARP 1, SIRT 1 and lowers levels of the proinflammatory cytokines IL-1β, IL-6, IL-8 and TNF-α. NAMR is an effective precursor of NAD^+^, but this may be due in part to its conversion into NAM by purine nucleoside phosphorylase (PNP). Thus, whereas both NAMR and NAM are equally effective in increasing levels of NAD^+^(P^+^) in mouse liver, NAMR raises liver [NAM] better than NAM itself [100]. Production of NAM from NAMR is further suggested from a study in one human subject [100] showing that NAMR increased NAM metabolite levels in plasma, urine and peripheral blood monocytes. NAMR, however, is three-fold more active than NAM in elevating levels of the ADP-ribose product of the NAD^+^-consuming enzymes PARP 1 and SIRT 1 [100]; an undesirable effect if PARP 1 is already activated in COVID-19. In another study [101], a similar dose of NAMR (1 g daily) was reported to increase [NAD^+^] in blood and [NAM] metabolites in blood and urine. Notably, NAMR lowered levels of IL-2, IL-5, IL-6 and TNF-α, but did not alter those of IFN-γ, IL-8, IL-12, MCP-1 and MIP-1B [101]. The NAMRH has been reported [102] to be a more effective NAD^+^ precursor than NAMR in mammalian cells and mouse tissues. It is a faster and stronger effector than NAMR and while it is partly oxidised to NAMR, it is not converted into NAM in mammalian cells, but undergoes such conversion in mice, as suggested by the elevation of [NAM] in plasma and liver. These mouse tissue changes are most likely the result of NAMRH conversion into NAMR. The authors [102] suggested that NAMRH-mediated production of NAD^+^ occurs via a previously unknown metabolic pathway independent of the NAMR kinase 1 (NRK 1) pathway. NAMRH also enhances PARP 1 activity in mouse kidney and potentiates that by cisplatin [102]. As far as I could ascertain, NAMRH has not been studied in humans. Thus, taken together, current evidence suggests that NAM should be the precursor of choice to replenish NAD^+^ levels in COVID-19 patients. A clinical trial of nicotinamide riboside (NAMR) in COVID-19 patients is currently underway in Denmark (NCT04407390), the outcome of which will be of interest in relation to the use of nicotinamide (NAM) and other NAD^+^ precursors discussed in the present hypothesis.

The COVID-19 therapeutic approach proposed in this paper, namely that involving PARP 1 inhibition and replenishing NAD^+^ by using its NAM and other precursors now represents one of the few remaining non-vaccine-based therapeutic options in the light of the Interim (huge) WHO Solidarity (randomised) Trial results, demonstrating the failure of Remdesivir, lopinavir, ritonavir, hydroxychloroquine and IFN-beta to influence overall mortality, initiation of ventilation and duration of hospital stay in hospitalised patients [103].

Further confirmation of upregulation of the PARP gene by AhR activation has very recently been reported [104] with a novel metabolic immune check-point, IL4I1 (IL-4-induced gene 1) (L-Phe oxidase). IL4I1 activates the AhR via KA and the Trp transamination product IPA. This indole metabolite is a strong PARP inducer and may act in part by conversion to KA ([104] via an unstable intermediate produced through interaction of the IPA enol form with reactive oxygen species (see [27] for details and references). The major role of KA in AhR activation is further suggested by IL4I1 increasing [KA] and not [Kyn] .

The discussion of the likely mechanism(s) of therapeutic efficacy of DEX in COVID-19 may have important implications for COVID-19 therapy and the mode of actions of prostaglandins. Thus, the potential role of the cytokine profile of COVID-19 in determining the efficacy or otherwise of glucocorticoids is worthy of investigation at the clinical and mechanistic levels and the prostaglandin activation of IDO induction by IFNs suggests that extending the focus of the proinflammatory effects of prostaglandins to tryptophan metabolism along the KP can open a new avenue of research on these inflammatory mediators.

Finally, It is almost certain that PARP 1 is strongly activated by COVID-19 and is responsible for death of patients. The partial success of some current therapies may be explained by the agents used not targeting PARP 1 directly, but are able to modulate a step(s) in the proposed sequence of events presented in the present paper with varying degrees of success. It is hoped that the above account will stimulate research efforts aimed at understanding the pathophysiology of COVID-19 viral infection and encourage clinicians to explore the potential therapeutic use of NAM as a PARP 1 inhibitor along standard clinical care and other therapies in the first instance and subsequently as an adjunct to stronger PARP 1 inhibitors and/or other drugs.

## Abbreviations

ACE 2: angiotensin-converting enzyme 2
ACMS: 2-amino-3-carboxymuconic acid-6-semialdehyde
ADP: adenosine diphosphate
AhR: aryl hydrocarbon receptor
BCL6: B Cell lymphoma 6 protein
COPD: chronic obstructive pulmonary disease
COX2: cyclooxygenase isoform 2
DEX: dexamethasone
ICU: intensive care use
IDO: indoleamine 2,3-dioxygenase
IFN: interferon
IL: interleukin
IL-1ra: Interleukin-1 receptor antagonist
KA: kynurenic acid
KP: kynurenine pathway
Kyn: kynurenine
MERS: Middle East respiratory syndrome
MHV: mouse hepatitis virus
NAM: nicotinamide
NAMR: NAM riboside
NAMRH: reduced NAMR
NEFA: non-esterified fatty acid
PA: picolinic acid
PARP: poly (ADP-ribose) polymerase
RSV: respiratory syncytial virus
SARS: severe acute respiratory syndrome
SIRT 1: Sirtuin 1 (silent mating type information regulation 2 homologue 1)
TCDD: 2,3,7,8-tetrachlorodibenzo-*p*-dioxin
Trp: *L*-tryptophan
XA: xanthurenic acid
3-HAA: 3-hydroxyanthranilic acid
3-HK: 3-hydroxykynurenine
